# Giant lipomatous tumor of the left back favoring atypical lipomatous tumor/well-differentiated liposarcoma: a case report

**DOI:** 10.1093/jscr/rjag707

**Published:** 2026-09-25

**Authors:** Ziling Zhang, Teng Fan, Xiaobo Yu, Qian Wang, Zhijie Zhang, Bin Wu

**Affiliations:** Department of Plastic Surgery, Xizang Autonomous Region People’s Hospital, No. 18 Linkuo North Road, Chengguan District, Lhasa 850000, China; School of Medicine, Xizang University, No. 36 Jiangsu Road, Chengguan District, Lhasa 850000, China; Department of Plastic Surgery, Xizang Autonomous Region People’s Hospital, No. 18 Linkuo North Road, Chengguan District, Lhasa 850000, China; School of Medicine, Xizang University, No. 36 Jiangsu Road, Chengguan District, Lhasa 850000, China; Department of Auricular Reconstruction, Plastic Surgery Hospital, Chinese Academy of Medical Sciences and Peking Union Medical College, No. 33 Badachu Road, Shijingshan District, Beijing 100144, China; Department of Pathology, Xizang Autonomous Region People’s Hospital, No. 18 Linkuo North Road, Chengguan District, Lhasa 850000, China; Department of Radiology, Xizang Autonomous Region People’s Hospital, No. 18 Linkuo North Road, Chengguan District, Lhasa 850000, China; Department of Plastic Surgery, Xizang Autonomous Region People’s Hospital, No. 18 Linkuo North Road, Chengguan District, Lhasa 850000, China

**Keywords:** atypical lipomatous tumor, well-differentiated liposarcoma, lipomatous tumor, giant tumor, case report

## Abstract

Giant lipomatous tumors of the trunk are rare and can pose diagnostic and reconstructive challenges. A 71-year-old man presented with a left back mass that had enlarged over 30 years, especially during the previous 2 years. The mass measured 56 × 54 × 28 cm clinically; computed tomography and magnetic resonance imaging showed a predominantly fat-containing thoracodorsal lesion measuring 27.1 × 18.6 cm on axial images, with septa and solid nodules but no definite muscle invasion. The tumor was excised under general anesthesia with flap reconstruction and weighed 10 kg. Histology showed mature adipocytes of variable size with scattered atypical stromal cells and lipoblasts. MDM2 immunostaining was negative, and molecular confirmation was unavailable; the findings favored an atypical lipomatous tumor/well-differentiated liposarcoma after clinicopathological correlation. Recovery was uneventful, with no recurrence at 9 months. Careful imaging, complete excision when feasible, and long-term surveillance are essential.

## Introduction

Atypical lipomatous tumor/well-differentiated liposarcoma (ALT/WDL) is a locally aggressive adipocytic neoplasm and represents a substantial subset of adult liposarcomas [1, 2]. Most lesions grow slowly and are painless, so diagnosis may be delayed until they become large enough to cause deformity, functional limitation, or operative difficulty. Cross-sectional imaging is central to preoperative planning because thick septa, nodular non-fatty components, and relationships to muscle and neurovascular structures help distinguish ALT/WDL from benign lipoma and guide excision [3]. Histopathology remains essential, and molecular testing for murine double minute 2 (MDM2) or cyclin-dependent kinase 4 (CDK4) amplification is often useful when morphology and immunohistochemistry are equivocal [4]. We report a giant left-back lipomatous tumor weighing 10 kg that clinically and morphologically favored ALT/WDL.

## Case report

**Figure 1:**
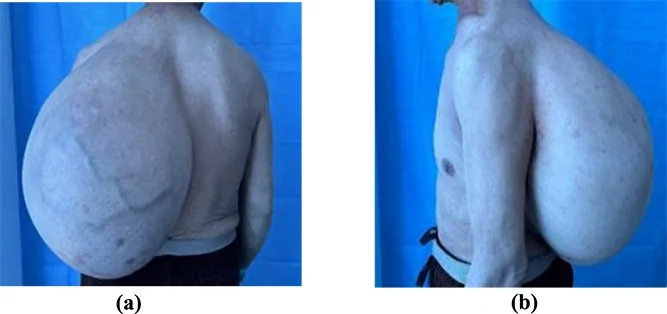
Preoperative clinical photographs of the giant left back tumor; (a) posterior view; (b) lateral view.

A 71-year-old man was admitted with a left back mass that had been present for 30 years and had enlarged rapidly during the previous 2 years. He had no remarkable past medical history. Examination showed a giant soft, well-circumscribed mass involving the left shoulder and back, extending from the suprascapular region to the lumbar region. The lateral borders reached the mid-axillary line and spine, and the surface showed superficial varicose veins. The clinical external size was ~56 × 54 × 28 cm (Fig. 1). No superficial lymphadenopathy was identified.

**Figure 2:**
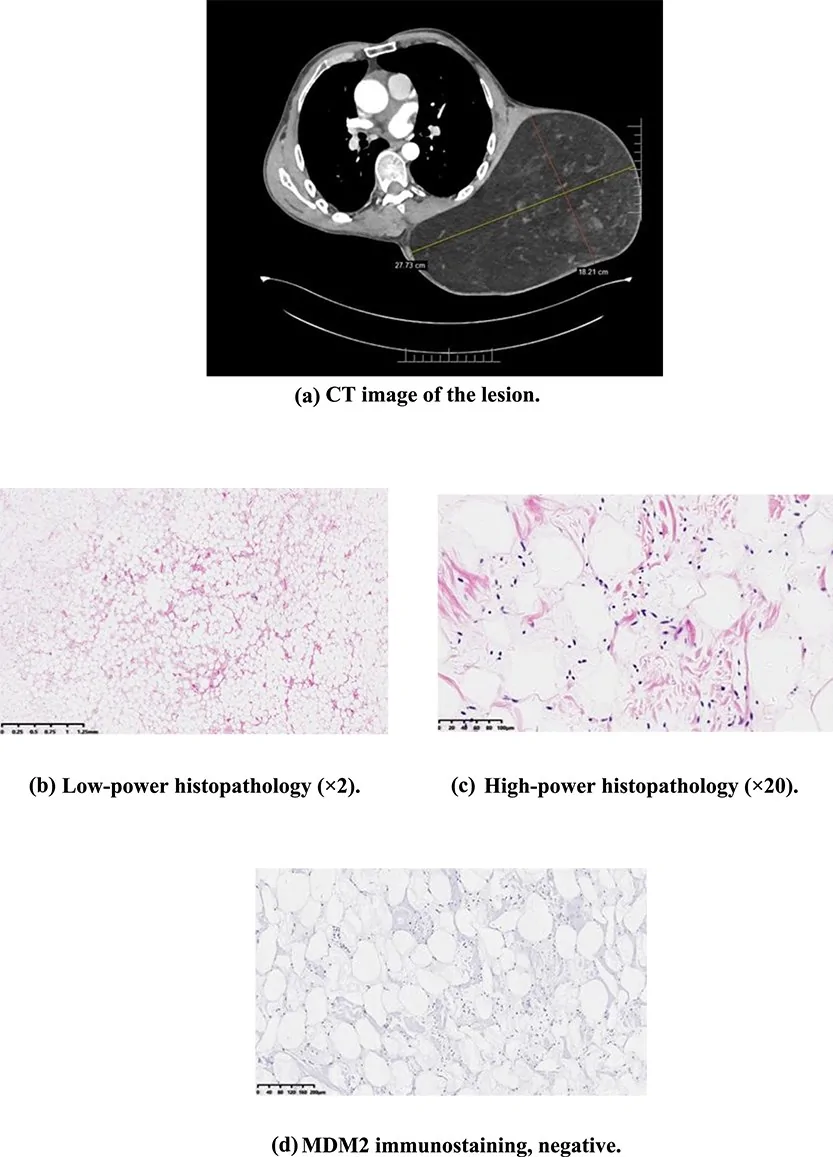
CT and histopathological findings of the tumor; (a) axial CT image showing a predominantly fat-density left thoracodorsal mass with septa and nodular soft-tissue components; (b) low-power histopathology; (c) high-power histopathology showing an adipocytic tumor with atypical stromal cells; (d) MDM2 immunostaining, negative.

Contrast-enhanced computed tomography (CT) showed a large fat-density mass in the left thoracodorsal region, measuring 27.1 × 18.6 cm on axial images. Internal solid nodules and fibrous septa were present, and adjacent muscles were displaced without definite invasion (Fig. 2a). Thoracoabdominal magnetic resonance imaging (MRI) confirmed a predominantly fatty mass with solid nodules and fibrous strands. Imaging suggested prominent paravertebral and thoracodorsal-region feeding vessels; the exact source artery was not specified. The difference between the clinical and imaging measurements reflected external 3D measurement versus the largest axial cross-section.

**Figure 3:**
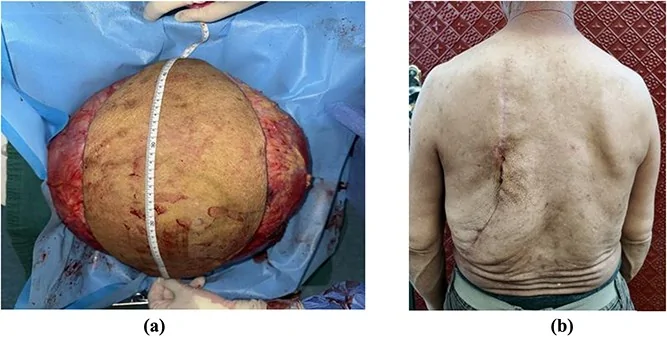
Resected specimen and postoperative follow-up; (a) gross specimen after gross complete excision; (b) Appearance at 9-month follow-up.

Preoperative hematological tests and tumor marker levels were within normal limits. After written informed consent, the patient underwent excision under general anesthesia with flap reconstruction. A spindle-shaped incision was made over the tumor surface. The encapsulated yellow tumor was dissected layer by layer, and the feeding vessels were ligated. The tumor was removed grossly intact and weighed 10 kg (Fig. 3a). Intraoperative blood loss was ~100 ml. Formal histological margin distance was not available, so the resection is described only as grossly complete.

Histology showed a lipomatous tumor composed of mature adipocytes of variable size with scattered atypical stromal cells and lipoblasts (Fig. 2b and c). Immunohistochemistry showed partial S-100 positivity, scattered weak P16 positivity, a Ki-67 index of ~2%, and negative MDM2 immunostaining (Fig. 2d). MDM2 fluorescence *in situ* hybridization, CDK4 immunohistochemistry, and 12q13–15 amplification testing were not available. After clinicopathological correlation, the findings favored ALT/WDL, but the lack of molecular confirmation was considered a diagnostic limitation. The drain was removed on postoperative Day 5 and sutures on Day 14. The incision healed well. No local recurrence or distant metastasis was observed at 9 months (Fig. 3b).

## Discussion

This case highlights three practical issues in managing giant superficial lipomatous tumors [5]. First, clinical size and imaging size may appear discordant when external 3D measurements are compared with the largest axial CT or MRI dimension. Reporting the measurement plane is therefore important, especially for very large deforming masses. Second, vascular mapping should be anatomically precise. In a giant thoracodorsal tumor, feeding vessels may arise from regional paravertebral, intercostal, lumbar, dorsal scapular, thoracodorsal, or other branches depending on tumor extent; a nonspecific assignment of arterial origin should be avoided unless confirmed by angiographic imaging.

Third, the diagnosis of ALT/WDL should not rely on MDM2 immunohistochemistry alone. ALT/WDL is typically composed of mature adipocytes with variation in cell size and atypical hyperchromatic stromal cells [1, 2]. MDM2 or CDK4 amplification testing can support distinction from benign lipoma and selected mimics, especially in older patients with large deep or recurrent tumors [4]. In the present case, morphology and imaging favored ALT/WDL, but negative MDM2 immunostaining and the absence of molecular testing require cautious wording. If archival tissue is available, MDM2 fluorescence *in situ* hybridization or equivalent 12q13–15 amplification testing would strengthen the final diagnosis.

Complete excision remains the main treatment for resectable ALT/WDL [6]. For giant superficial tumors, preoperative imaging should define the capsule, muscle invasion, and main vessels; intraoperative dissection should balance complete removal with preservation of major neurovascular structures and soft-tissue coverage. For retroperitoneal or other deep tumors, multidisciplinary planning and extended resection may be required [7]. Because margin distance was not documented here, margin status should not be described as pathologically negative. Radiotherapy is generally reserved for selected high-risk, unresectable, or margin-positive situations, and its benefit varies by anatomical site [8–10]. Conventional chemotherapy has limited value for ALT/WDL unless dedifferentiated or metastatic disease is present, while molecularly targeted approaches remain investigational or context dependent [11, 12].

Although ALT/WDL has low metastatic potential, local recurrence and dedifferentiation can occur, particularly after incomplete excision or in anatomically constrained sites [13]. This patient remained disease-free at 9 months, but longer surveillance with periodic clinical examination and imaging is needed. The key lessons are to document imaging measurements precisely, avoid overinterpreting unconfirmed vascular anatomy, acknowledge the MDM2-negative/molecularly unconfirmed diagnostic limitation, and report resection status only when pathological margin data support it. This case was reported in line with the SCARE guideline [14].

## Declarations

Ethical approval was waived because this single-patient case report did not constitute human-subjects research according to institutional policy.

Written informed consent was obtained from the patient for publication of this case report and accompanying images.

Research registration was not applicable.

During manuscript preparation, the authors used ChatGPT only to improve language and readability; no AI tool was used for data collection, data analysis, image generation, or clinical decision-making. The authors reviewed and edited all AI-assisted content and take responsibility for the final manuscript.

## References

[1] Arora K, Rosenberg AE. Lipomatous neoplasms of soft tissue: a contemporary review. Adv Anat Pathol 2025;32:147–56. 10.1097/PAP.0000000000000468 39434553

[2] Mashima E, Sawada Y, Nakamura M. Recent advancement in atypical lipomatous tumor research. Int J Mol Sci 2021;22:994. 10.3390/ijms22030994 PMC7863944 33498189

[3] Noebauer-Huhmann IM, Vanhoenacker FM, Vilanova JC et al. Soft tissue tumor imaging in adults: whole-body staging in sarcoma, non-malignant entities requiring special algorithms, pitfalls and special imaging aspects. Guidelines 2024 from the European Society of Musculoskeletal Radiology (ESSR). Eur Radiol 2025;35:351–9. 10.1007/s00330-024-10897-z PMC11631817 39030374

[4] Kilpatrick SE . Atypical lipomatous tumor/well differentiated liposarcoma and related mimics with updates. When is molecular testing most cost-effective, necessary, and indicated. Hum Pathol 2024;147:82–91. 10.1016/j.humpath.2023.12.005 38135062

[5] Silistreli OK, Durmus EU, Ulusal BG et al. What should be the treatment modality in giant cutaneous lipomas? Review of the literature and report of 4 cases. Br J Plast Surg 2005;58:394–8. 10.1016/j.bjps.2004.09.005 15780237

[6] Rauh J, Klein A, Baur-Melnyk A et al. The role of surgical margins in atypical lipomatous tumours of the extremities. BMC Musculoskelet Disord 2018;19:152. 10.1186/s12891-018-2053-3 PMC5960141 29776450

[7] Asencio Pascual JM, Fernandez Hernandez JA, Blanco Fernandez G et al. Update in pelvic and retroperitoneal sarcoma management: the role of compartment surgery. Cir Esp (Engl Ed) 2019;97:480–8. 10.1016/j.ciresp.2019.06.011 31521244

[8] Wiltink LM, Spalek MJ, Sangalli C et al. The role of standard and novel radiotherapy approaches in management of retroperitoneal sarcomas. Eur J Surg Oncol 2023;49:1111–4. 10.1016/j.ejso.2022.08.029 36115783

[9] Lawless A, Zhou DD, McDonough J et al. The role of radiation therapy in the management of primary retroperitoneal sarcoma: a systematic review and clinical practice guidelines from the Australia and New Zealand Sarcoma Association. Cancer Treat Rev 2023;120:102620. 10.1016/j.ctrv.2023.102620 37657126

[10] Salerno KE, Baldini EH. Role of radiation therapy in retroperitoneal sarcoma. J Natl Compr Canc Netw 2022;20:845–9. 10.6004/jnccn.2022.7035 35830885

[11] Lee TY, von Mehren M. Novel pharmacotherapies for the treatment of liposarcoma: a comprehensive update. Expert Opin Pharmacother 2024;25:2293–306. 10.1080/14656566.2024.2427333 39535168

[12] Martin-Broto J, Martinez-Garcia J, Moura DS et al. Phase II trial of CDK4/6 inhibitor palbociclib in advanced sarcoma based on mRNA expression of CDK4/CDKN2A. Signal Transduct Target Ther 2023;8:405. 10.1038/s41392-023-01661-8 PMC10598203 37875500

[13] von Mehren M, Kane JM, Agulnik M et al. Soft tissue sarcoma, version 2.2022, NCCN clinical practice guidelines in oncology. J Natl Compr Canc Netw 2022;20:815–33. 10.6004/jnccn.2022.0035 PMC10186762 35830886

[14] Kerwan A, Al-Jabir A, Mathew G et al. Revised Surgical CAse REport (SCARE) guideline: an update for the age of artificial intelligence. Premier J Sci 2025;10:100079.

